# Induced Pluripotent Stem Cells as a Tool to Decipher the Normal and Abnormal Development of the Esophagus and Trachea from Normal Morphogenesis to Esophageal Atresia, Tracheomalacia, and Laryngo–Tracheal Clefts

**DOI:** 10.3390/cells15050448

**Published:** 2026-03-03

**Authors:** Yuxuan Zhang, Anu David, Alireza Nemati, Christophe Faure

**Affiliations:** 1 Esophageal Development and Engineering Laboratory, CHU Sainte-Justine Research Centre, Université de Montréal, 3175 Chemin de la Côte-Sainte-Catherine, Montréal, QC H3T 1C5, Canada; yuxuan.zhang.1@umontreal.ca (Y.Z.); alireza.nemati.1@umontreal.ca (A.N.); 2 Department of Pediatrics, Université de Montréal, 3175 Chemin de la Côte-Sainte-Catherine, Montréal, QC H3T 1C5, Canada; 3 Esophageal Atresia Clinic and Division of Pediatric Gastroenterology Hepatology and Nutrition, CHU Sainte-Justine, 3175 Chemin de la Côte-Sainte-Catherine, Montréal, QC H3T 1C5, Canada

**Keywords:** endoderm, mesoderm, anterior foregut, esophagus, trachea, esophageal atresia, tracheomalacia, laryngeal–tracheal clefts, induced pluripotent stem cells

## Abstract

The development of the esophagus and trachea following the septation of the anterior foregut is a highly regulated process involving bidirectional communication between the endoderm and mesoderm. Signaling pathways such as the Bone Morphogenetic Protein family, Wnt/β-catenin, Sonic Hedgehog, and Fibroblast Growth Factor family mediate this complex crosstalk to induce the dorsal-ventral patterning of the anterior foregut as well as lineage specification. Even though the mechanisms are not fully understood, dysregulation of signaling pathways may lead to congenital malformations such as tracheomalacia, laryngeal–tracheal clefts and multiple types of esophageal atresia with/without tracheoesophageal fistula (EA/TEF). Human induced pluripotent stem cells (iPSCs) provide a robust *in vitro* platform to monitor the normal and abnormal development of esophagus and trachea and to understand the roles of the endoderm and mesoderm during anterior foregut development. Recent studies have demonstrated that direct differentiation of iPSCs into epithelial and mesenchymal lineages can recapitulate the key stages of foregut development. In this regard, in the current paper, we review the signaling pathways involved in the development of organs deriving from the anterior foregut as well as the roles of the endoderm and mesoderm revealed by previous studies. Furthermore, we discuss the use of iPSCs as a valuable model for investigating the bidirectional communications between the endoderm and mesoderm, which can broaden our knowledge and understanding of the critical mechanisms leading to normal and abnormal development of the esophagus and trachea.

## 1. Introduction

During the early stages of mammalian embryogenesis, the esophagus and trachea develop from the dorsal and ventral sides of the anterior foregut, respectively [1,2,3]. Dorsally, the esophagus matures developmentally, showing the presence of stratified squamous epithelium layered with basal and suprabasal cells expressing the transcription factor SOX2, and the mesenchyme with a well-organized alignment of smooth muscle and skeletal muscle [1,4,5]. Ventrally, the tracheal epithelium, expressing another transcription factor, NKX2.1, comprises multiple cell types necessary for respiratory function, including basal cells and the apical ciliated population [6,7,8,9,10,11,12]. Furthermore, cartilage and smooth muscle are the principal tracheal mesenchymal lineages [5,13]. These specifications originate in the anterior foregut region at around 4–5 weeks during human embryonic development, including the generation of the anterior–posterior axis, followed by dorsal–ventral patterning [3,14,15]. Budding of the trachea primordium and the separation between trachea and esophagus are induced and regulated by a complex coordination between multiple signaling pathways, including bone morphogenetic proteins (BMPs), canonical and non-canonical wingless/integrated signaling (WNT), hedgehog, and fibroblast growth factor signaling (FGF). These signaling molecules play a critical role in the bi-directional communication between the anterior foregut endoderm and mesoderm, in a series of molecular events [16,17,18,19,20].

Dysregulation of this accurately mediated crosstalk may lead to abnormal anterior foregut compartmentalization, leading to esophageal and tracheal malformations in newborns, such as congenital tracheomalacia, laryngeal–tracheoesophageal clefts, and multiple types of esophageal atresia with/without tracheoesophageal fistula (EA/TEF), occurring in 1 in 3000 newborns [21,22,23,24,25,26]. However, the mechanisms underlying this malformation are poorly understood. From a clinical perspective, esophageal atresia remains a condition associated with significant morbidity and mortality despite advances in neonatal surgery and intensive care. Population-based clinical studies demonstrate substantial variability in associated anomalies and long-term outcomes across regions and healthcare systems, underscoring the complex and multifactorial nature of the disease. Long-term follow-up data further highlight persistent respiratory, gastrointestinal, and nutritional complications extending into childhood and beyond. These clinical observations reinforce the need for a deeper understanding of the developmental mechanisms underlying esophageal and tracheal malformations [27,28]. Therefore, a complete temporal–spatial map describing these molecular events between the mesoderm and endoderm is required to understand the normal and abnormal compartmentalization of the anterior foregut. Studies involving animal models have provided valuable information in this regard [3,17,22,29,30,31,32,33,34,35,36,37]. In the past decade, stem cells, especially, induced pluripotent stem cells (iPSCs), have been widely used to understand embryonic development as well as to create disease models [4,21,38,39]. Directed differentiation of iPSCs has been performed to generate esophageal- and respiratory-like epithelium and mesenchyme, monitoring different crucial developmental stages including development of the anterior foregut endoderm and mesoderm [3,4,21,38,40,41,42,43,44,45]. Furthermore, using EA/TEF patient-derived iPSCs could be a valuable tool to understand the underlying mechanisms involved during esophageal and tracheal development, mimicking a patient-specific pathogenic environment to understand the dysregulated parts of the complex interaction network [21]. Therefore, the focus of this review is to highlight the studies performed over the past few decades that have tried to decipher the mechanisms and pathways involved during the early stages of embryonic development. In this regard, we discuss anterior foregut development and compartmentalization into the esophagus and trachea, including the communications occurring between the endoderm and mesoderm. Furthermore, we outline the molecules, factors, and pathways involved in this crucial development stage, using iPSCs as a tool.

## 2. Compartmentalization and Separation of Anterior Foregut and Maturation of Organ-Specific Epithelium and Mesenchyme

**Figure 1:**
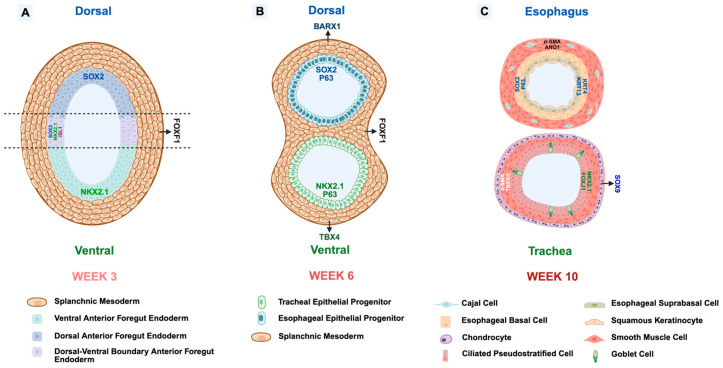
Tracheoesophageal separation and the specification of epithelium and mesenchyme. (**A**) In the anterior foregut of week 3 human embryos, SOX2 expression is in the endoderm on the dorsal side and NKX2.1 expression is in the endoderm on the ventral side, with the surrounding splanchnic mesoderm expressing FOXF1. The endodermal cells at the expected dividing area co-express SOX2, NKX2.1, and ISL1. (**B**) At week 6, the simple columnar structure is established in the esophageal epithelium and tracheal epithelium, with the presence of SOX2 and P63 in esophageal, and NKX2.1 and P63 in tracheal epithelium. (**C**) At week 10, the esophageal epithelium specifies into a stratified structure containing basal, suprabasal, and apical layers with squamous keratinocytes expressing SOX2, P63, KRT4, and KRT13. The esophageal mesenchyme contains smooth muscle and Cajal cells, expressing α-SMA and ANO1, respectively. The tracheal epithelium specifies into ciliated pseudostratified cells and goblet cells, and the smooth muscle is surrounded by cartilage rings in the mesenchyme.

The primitive streak, which is established during gastrulation, initiates the embryonic tissue specification. In a series of early morphogenesis events, such as the epithelial-to-mesenchymal transition (EMT), mesendoderm differentiates into lateral plate mesoderm and definitive endoderm lineages, forming a gut tube [3,14,15]. By week 3 of human embryonic development, the anterior–posterior axis is established to generate the foregut, midgut, hindgut, and further regionalization into anterior and posterior foregut, evidenced by region-specific gene expression [14,15]. Dorsal–ventral patterning of the foregut is then initiated by a precisely modulated signaling network between the definitive endoderm and the splanchnic mesoderm [3,14]. The ventral side is the site where the future tracheal primordium forms, indicating its respiratory fate. The endodermal cells in the ventral anterior foregut form a pool of tracheal epithelial progenitor cells after the separation of the trachea and esophagus [1]. The esophageal fate is located on the dorsal side of the anterior foregut, and the endodermal cells are the precursors of the esophageal epithelial progenitor cells (Figure 1A) [1]. During week 4 of human development, the tracheal primordium emerges and initiates the physical separation into the dorsal esophagus and a ventral trachea [2,3,46]. At this stage, the endodermal cells on the dorsal side express SOX2 and those on ventral side express NKX2.1. Studies involving mouse embryos (E9.5) showed that the cells at the boundary co-express Sox2, Nkx2.1, and Isl1 [3,22,29,30]. To separate into two tubes, these boundary endodermal cells constrict and fuse due to the compressive force generated in the surrounding mesenchyme, which expresses Foxf1, followed by the generation of cell polarity [47]. A study using a xenopus model revealed the role of Rab11a recycling endosome in modulating the fusion of the apical epithelial cells in the anterior foregut, by recruiting Vangl-Celsr complexes, which are responsible for cell polarity during the separation [48]. In addition, the compressive force generated in the mesenchyme has been proven from the migration of mesenchymal cells towards the epithelium, but not from differential proliferation in the mesenchyme [49].

After completing the anterior foregut compartmentalization, the growth of the human esophagus and trachea becomes more independent, and the lengthening of the esophagus is faster. After the separation from the trachea, the esophageal epithelium structure is only a layer of simple columnar epithelial cells [5]. This single-layer epithelium persists until week 6 and continuous SOX2 expression maintains the esophageal fate. Then, with the expression of P63 and KRT5, basal progenitor cells initiate and continuously proliferate to support the mucosa. These cells are the direct precursors of the basal keratinocytes, which keep their stem cell potential for repairing and generating suprabasal cells (Figure 1B) [1,4]. The specification of suprabasal cells, which will become the middle layer of the epithelium, is observed by week 8, with the identity of the non-cornified stratified squamous mucosa showing the expression of KRT4 and KRT13 [1,5]. Furthermore, the suprabasal cells possess improved regeneration characteristics and become a barrier, which is distinctly different from the intestinal columnar progenitors. These cells act as a pool of differentiating epithelial cells, which finally develop into a fully differentiated superficial squamous layer by week 10 to resist mechanical and chemical stress (Figure 1C) [1,4]. The superficial squamous keratinocytes, like their precursors, are resistant to acidic and physical stimulation and highly regenerative to renew damaged cells [4,5]. Similar to the esophageal epithelia, the simple columnar epithelium is also the main structure in early tracheal epithelia at around week 5, but develops from the ventral anterior foregut endoderm with a committed respiratory fate [5]. Beginning in week 6, the tracheal basal progenitor cells are observed, with the expression of P63, KRT5, and NKX2.1. Similar to esophageal basal cells, these cells in the basal layer are also primary progenitors of the specified tracheal epithelial cells, as proved by clonal lineage labelling and single-cell molecular analysis [10,12,50,51]. However, the surface markers and the cytokeratin secreted by trachea basal cells support the growth of pseudostratified epithelium but not stratified squamous epithelium (Figure 1C) [1,52]. As a reservoir of stem cells, these basal cells possess and retain the capacity for self-renewal during and after the specification and maturation of the tracheal epithelium [53]. The morphogenesis of the ciliated cells initiates in week 7 followed by active ciliation by week 12 [6]. The establishment of ciliated cells is required for the postnatal ability to mechanically remove mucus and external particles upward into the pharynx [6,7]. The secretory cells are important for secreting mucus containing mucin, which forms a superficial liquid and mucous layer to entrap particles and pathogens [11]. Goblet cells, which constitute a significant proportion of the human tracheal secretory cells, develop from week 10, followed by club cells that develop later [11,12,54]. Furthermore, pulmonary neuroendocrine cells, pulmonary ionocytes, and brush cells represent a minor proportion of the tracheal epithelial cells; however, they are crucial for chemical modulation and sensation in the tracheal epithelium [8,9,10,54]. Following the tracheoesophageal separation, the immature tracheal epithelium and esophageal epithelium are surrounded by their own mesenchyme, which will differentiate into smooth muscles for both the esophagus and trachea, and the discontinuously arranged cartilage rings become specific for trachea [5,13,54,55]. In humans, skeletal muscle surrounds the proximal third of the esophagus, and smooth muscle surrounds the distal third, while the middle third of the esophagus is covered by a combination of skeletal muscle and smooth muscle [5]. Different from the mouse esophagus, the human esophagus has a thicker epithelium containing 20–30 layers of cells but without a layer of keratin on top of the epithelium. However, a number of ciliated esophageal epithelial cells are present in both human and mouse embryos and disappear after birth [5].

Since 2015, advances in the field of bioinformatics, including machine learning, have been utilized to demonstrate the lineage trajectory and reveal the cellular events during mouse and human embryonic development [3,56,57]. Yang et al. used Chromium, Visium, and Codex to visualize the spatiotemporal process of the human embryonic esophageal mucosa development, based on data derived from mid-first trimester to mid-second trimester aborted fetuses [56]. They identified the process of esophageal mesenchymal cells’ development, simultaneously with waves of epithelial differentiation. Four types of fibroblasts were identified; Fib_1 cells are responsible for epithelium proliferation regulation and differentiation; Fib_2 cells express collagen, fibrillin, and proteoglycan, helping to support the esophageal structure; Fib_3 cells, crucial for neural and muscle function, and myofibroblasts were confirmed to be differentiated from an early fibroblast progenitor cluster, which is abundant until E59 [56]. After E80, the fibroblasts responsible for the vascularization and the function of blood vessels also give rise to adventitial fibroblasts [56]. Furthermore, the group revealed that the esophageal mesenchymal cell lineages responsible for peristalsis, smooth muscle cells, and interstitial cells of Cajal are independently established during the mid-first trimester of the human fetus [56]. Simultaneously, the progenitors of pericytes are present by E47; researchers identified this cell population as a self-maintaining progenitor pool that gives rise to three types of pericytes, responsible for cell differentiation, angiogenesis, and muscle contraction, respectively, after E80 [56].

## 3. Key Transcription Factors Involved in Tracheal–Esophageal Separation and Development

### 3.1. SOX2

**Figure 2:**
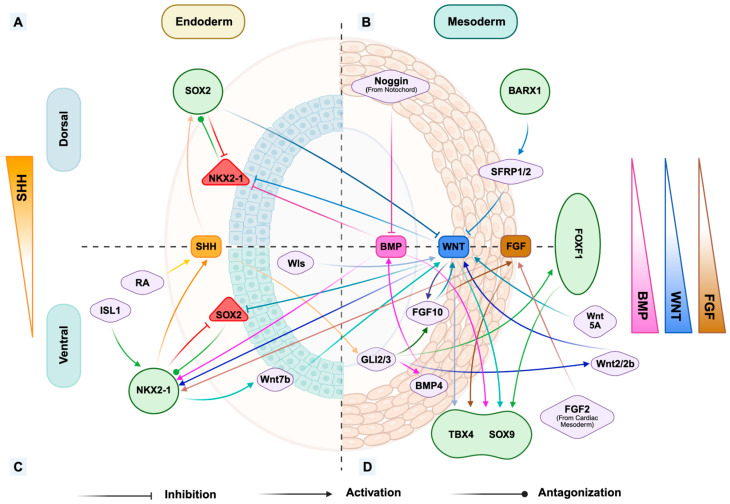
Interactions between mesoderm and endoderm during dorsal–ventral patterning and trachea and esophageal development modulated by the crosstalk between various molecular signaling pathways. (**A**,**B**) Dorsal mesodermal transcription factor BARX1 modulates SFRP1/2 to inhibit the mesodermal WNT secretion towards dorsal endoderm. WNT interacts with notochord noggin to inhibit mesodermal BMP which further represses NKX2.1 (respiratory fate) in the dorsal endoderm. The absence of NKX2.1 results in SOX2 (esophageal fate) increase. Furthermore, SHH secretion in endoderm improves esophageal fate on the dorsal side. (**C**,**D**) NKX2.1 expression in the ventral endoderm is induced by a network of multiple signaling pathways. Endodermal SHH secretion modulated by retinoic acid activates the downstream GLI family, BMP, WNT, and FGF signaling due to the mesodermal secretion of BMP4, Wnt2/2b, and FGF10 towards the endoderm to stimulate NKX2.1 expression. FGF10 is modulated by WNT and FGF2, which is secreted by the cardiac mesoderm, stimulating FGF activation. The upstream transcription factor, ISL1, is also necessary for NKX2.1 expression. The activation of non-canonical WNT by Wnt5A and FGF10 represses SOX2 esophageal programming on the ventral side, which further improves NKX2.1 expression and antagonism against SOX2. NKX2.1 induces the epithelial paracrine secretion of Wnt7b to improve the specification of the adjacent mesenchymal cells into cartilage expressing SOX9. Wls, a ligand of WNT, is necessary for TBX4^+^ respiratory mesenchymal development. Furthermore, FGF10 autocrine secretion is important for ventral mesodermal development and the activation of GLI2/3 by endodermal SHH. SHH stimulates FOXF1 expression in mesoderm which induces the physical separation between trachea and esophagus and improves tracheal cartilage development. Lines and arrows show the activating and inhibitory interactions between signaling pathways: SHH (orange and yellow), BMP (rose), WNT (blue and cyan), and FGF (brown).

As a member of the SOX family, SOX2 is a crucial transcription factor involved in the specification of the progenitor cells during the early stage of embryogenesis. In the anterior foregut endoderm, SOX2 expression is principally restricted to the dorsal domain due to the repression of BMP signaling. Acting as an antagonist to NKX2.1, which determines the ventral respiratory fate, the future identity of the dorsal side towards the esophagus is enhanced. SOX2 has been proven to inhibit the canonical WNT signaling in the dorsal anterior foregut (Figure 2A,B) [58]. The deletion of *Sox2* in mouse embryos leads to the upregulation of the WNT target genes, and the overexpression of *Sox2* results in the inhibition of WNT signaling and further inhibition of Nkx2.1 expression [4,59]. On the border between the dorsal and ventral sides of the mouse anterior foregut, Sox2 is co-expressed with Nkx2.1 and Isl1 in the midline epithelial cells before the physical separation between trachea and esophagus occurs [3,22,29,30]. Following anterior foregut compartmentalization, Sox2 expression is maintained in the developing esophageal epithelium [29]. SOX2 is also important for the survival of the esophageal epithelial progenitors. Its aberrant downregulation leads to the apoptosis and the malformations of the esophagus, including esophageal agenesis, indicating the parallel roles of SOX2 in maintaining the cell fate and cell viability [60,61]. Furthermore, as a transcription factor that can define the fate of stratified squamous epithelial cells, Sox2 binds to the enhancers of squamous genes and coordinates with p63 to program the stratified squamous fate in mice. This coordination ensures the normal differentiation of the esophageal epithelium [60,61]. The effect of Sox2 in this pathway is time- and dose-dependent, and precisely controlled Sox2 expression is required for normal esophageal epithelium development [22,62]. Its overexpression results in the premature proximal basal cell proliferation, and insufficient Sox2 leads to the loss of esophageal fate and the abnormal expression of respiratory markers [22,62]. The limited BMP activation in the esophagus allows the continuous SOX2 expression to maintain the esophageal fate, with improvement of the specification of the epithelium [4]. During the maturation of the specified esophageal epithelium in humans, SOX2 expression keeps inhibiting the expression of CDX2, which can result in intestinal-like columnar differentiation, to maintain the identity of stratified squamous epithelial cells and prevent their trans-differentiation [58]. The dysregulation of SOX2 in the anterior foregut and in the esophagus plays a crucial role in the development of EA/TEF. In mouse embryos, downregulated Sox2 expression affects the establishment of the dorsal domain and induces ectopic expression of Nkx2.1, which results in failure of tracheoesophageal separation. In a study with *Sox2* knockdown mouse embryo mutants, about 60% of the mutants developed EA/TEF [22]. In humans, loss of SOX2 function has been proven to lead to the development of anophthalmia–esophageal–genital (AEG) syndrome including the clinical EA/TEF [23]. Insufficiency of SOX2 after the tracheoesophageal separation can result in a combination of esophageal-like and tracheal-like epithelium, together with a fistulous-like tissue [21,22].

To conclude, as a principal dorsal transcription factor, SOX2 expression is modulated by and coordinates with multiple signaling pathways to maintain the dorsal–esophageal fate by antagonizing the respiratory marker NKX2.1 to improve and induce the survival, proliferation, and differentiation of the esophageal epithelium (Figure 2A,B) [4,18,59,61]. Loss of SOX2 before the tracheoesophageal separation is considered to be the most crucial effect leading to EA/TEF, because of the failed establishment of dorsal fate and the failure of the separation related to the ectopic expression of NKX2.1 [21,22,23].

### 3.2. NKX2.1

NKX2.1 is a crucial transcription factor involved in the respiratory fate on the ventral side of the anterior foregut. Wnt2/2b activates the canonical Wnt/β-catenin signaling in endoderm and induces the expression of Nkx2.1 (Figure 2C,D), which is required to determine the tracheal fate of these cells [16]. The stabilization of Nkx2.1 expression and the ventral transcriptional program is regulated by the coordination of BMP and Wnt signaling activation [18]. Isl1, an upstream transcription factor, is necessary to activate the expression of Nkx2.1 in the midline between the dorsal and ventral sides of the anterior foregut endoderm (Figure 2C) [29]. This process leads to normal tracheoesophageal separation [29]. In parallel, Nkx2.1 antagonizes the expression of Sox2 in the ventral anterior foregut endoderm to inhibit the development of dorsal fate (Figure 2C) [63]. Morphogenesis regulator Efnb2 has been proven to be inhibited by Nkx2.1 on the ventral side during the tracheoesophageal separation, to establish the boundary of the future tracheal epithelium [64]. Following the physical tracheoesophageal separation, Nkx2.1 is the principal transcription factor responsible for the establishment and specification of the tracheal epithelium [65]. The expression of Nkx2.1 in the tracheal epithelium is crucial for maintaining the progenitors’ fate [65]. Its expression improves the expression of genes required for the further specification of ciliated and secretory cells, while inhibiting intestinal-like cells’ induction programs [65]. Nkx2.1 deficiency significantly affects the proliferation and the specification of the tracheal epithelial progenitors [66]. Furthermore, Nkx2.1 expression in the epithelium is important in regulating the differentiation of mesenchymal cells [59]. NKX2.1 induces the secretion of epithelial paracrine ligands, including Wnt7b and Shh, which is required for gene expression during the specification of the adjacent mesenchymal cells into cartilage (Figure 2C,D) [59]. In mice, the lack of Nkx2.1 has been proven to induce mesenchymal differentiation into smooth muscle instead of cartilage [66]. Before the separation, loss or insufficiency of Nkx2.1 leads to failure of the establishment of the ventral domain and the border between the dorsal and ventral anterior foregut endoderm. This directly results in the development of EA/TEF [59] and the expression of Sox2 replaces it in anterior foregut without normal dorsal–ventral patterning [66]. After the tracheoesophageal separation, the downregulation of Nkx2.1 results in aberrant epithelial and mesenchymal patterns, and further abnormal junctions between these two layers leads to the formation of a fistula [29]. Ectopic expression of NKX2.1 on the dorsal side has been observed in patient-derived anterior foregut endoderm, indicating its roles in determining the fate of the epithelium of the fistulous tract [21].

To conclude, NKX2.1 is modulated by WNT and BMP signaling and is crucial for initiating the tracheoesophageal separation and further tracheal maturation, including the specification of the tracheal epithelium and mesenchyme, by antagonizing SOX2 (Figure 2C,D) [16,18,29,59,63,65,66]. Dysregulation of NKX2.1 expression before the anterior foregut compartmentalization results in EA/TEF, and its dysregulation after the separation may complicate the clinical characteristics of EA/TEF [21,29,59,66]. Furthermore, NKX2.1 mutation has also been clinically reported in patients with tracheomalacia [67].

### 3.3. FOXF1

As a downstream target of Sonic Hedgehog (SHH) signaling, the expression of FOXF1 is first observed in the lateral plate mesoderm. Its presence is crucial for the establishment of the splanchnic mesoderm surrounding the anterior foregut endoderm [68]. The expression of FOXF1 indicates the activity of SHH signaling, especially in the ventral mesoderm before the foregut compartmentalization initiates [69]. Furthermore, Foxf1 precisely controls the activity of WNT signaling in the mouse embryonic mesenchyme. Together with the secretion of the extracellular matrix (ECM), it stabilizes the structure of the anterior foregut before the physical separation between trachea and esophagus [70]. Furthermore, the high expression of Foxf1 in the ventral mesoderm promotes its interactions with the ventral endoderm, specific to the respiratory domain, and further supports the development of lung buds [71]. Therefore, the dysregulation of Foxf1 expression in this stage leads to the malformations of the mouse anterior foregut, including EA/TEF [69]. In the mouse esophagus, after the separation from the trachea, *Foxf1* is necessary for the specification of the mesenchyme into smooth muscle, and its deletion leads to a thinner esophageal mesenchyme [72]. The dysregulation of Foxf1 also results in an abnormal esophageal epithelium. Like the esophagus, the mesenchyme’s specification into smooth muscle and cartilage rings in the trachea requires sufficient expression of Foxf1 [24,72]. Furthermore, the proliferation of the tracheal epithelium, which only expresses Nkx2.1 but not Sox2, needs Foxf1 from the surrounding mesenchyme [72]. A modest downregulation from the early stage of embryogenesis is enough to lead to anterior foregut malformations by affecting the development of the mesenchyme (Figure 2D) [69]. Complete loss of *Foxf1* directly results in the death of mouse embryos [68]. EA/TEF development can be caused by insufficient expression of Foxf1 leading to abnormal epithelial–mesenchymal interactions during the foregut compartmentalization [69]. Insufficient Foxf1 can cause abnormal tracheal and lung development with disordered respiratory mesenchyme [69]. Furthermore, the lack of Foxf1 in mouse embryos has been proven as a reason for tracheomalacia development because of the reduced secretion of downstream ligand Rspo2 [24]. However, studies also revealed that its overexpression in the esophageal squamous epithelium may lead to the development of columnar-like cells, characterizing Barrett’s esophagus [73].

## 4. Pathways Associated with Anterior Foregut Development and Tracheoesophageal Specification

### 4.1. BMP Signaling

Bone morphogenetic proteins (BMPs) are members of the TGF-β superfamily. A well-regulated BMP signaling is crucial for the developmental events during embryogenesis, including the appropriate development and compartmentalization of the anterior foregut. The antagonism of BMP signaling by Noggin is important for the development of the dorsal anterior foregut, esophagus, and the activation by the ligand Bmp4 is crucial for the development of the ventral anterior foregut and further trachea [32,33,74,75]. Multiple *in vitro* studies have proved that the inhibition of BMP improves the esophageal fate of the differentiating cells and BMP activation leads to a respiratory fate (Figure 2C) [21,43]. During gastrulation, Bmp4, a key factor for ventralization is expressed [74]. As an activator of BMP signaling, Bmp4 is required to initialize the pathway during the airway morphogenesis [75]. Bmp4-lacZ reporter mouse embryos indicated that the initial Bmp4 expression in the mesenchyme begins on both the left and right sides of the early anterior foregut, before visible lung budding appears. This reveals that BMP signaling is involved in respiratory fate patterning during a very early stage before the dorsal–ventral endodermal compartmentalization [32]. Bmp4 generated from the ventral side of the mesenchyme surrounding the anterior foregut endoderm leads to the tracheoesophageal separation and ventral development [76,77]. After the generation of different types of tracheal epithelial cells, including pseudostratified columnar epithelial cells, basal cells, and ciliated cells, Bmp4 is expressed in the distal lung endoderm [32]. Bmp4 has also been proven to be required for mesodermal development (Figure 2D) [78]. The expression of Brachyury T (a T-box transcription factor), which is critical for inducing the middle primitive streak, is absent in all the homozygous Bmp4^tm1blh^ genotyped mouse embryos, indicating abnormal early mesoderm development [78]. Furthermore, the activation of BMP signaling by the expression of Bmp4 is necessary for the process inducing epithelial–mesenchymal transition (EMT), which generates a large proportion of mesenchyme during anterior foregut organogenesis [38,45,79]. Furthermore, BMP4 disruption has been revealed to be related to the development of trachea–laryngeal clefts in human and mouse embryos [80]. Bmpr1a and Bmpr1b are the two main receptors of BMP signaling [18]. In mouse embryos with the inactivation of both receptors, the normal tracheoesophageal separation did not happen, with a single tube showing dorsal/esophageal characters, such as the expression of p63 and Sox2 and the absence of Nkx2.1 expression until E11.5 [18]. This indicates a complete loss of the respiratory fate after blocking BMP signaling. The downstream proteins of BMP signaling such as Smad1/5/8, which are responsible for the transduction of Bmp4 signals from the cell membrane to the nucleus, have been shown to be crucial in the airway development [81]. The downregulation of these proteins results in inhibition of the proliferation and the differentiation of the airway epithelial cells [81]. When genes encoding Smad1/5/8 proteins are deficient in the ventral anterior foregut endoderm, Bmpr1a and Bmpr1b are abolished [18].

Noggin, a BMP antagonist encoded by the *Nog* gene, is expressed in the notochord and dorsal anterior foregut to inhibit BMP signaling and promote the dorsal and esophageal fate by inducing the expression of Sox2 (Figure 2A,B) [32,33,82,83]. The source of the secretion of Noggin is crucial for normal dorsal–ventral patterning and tracheoesophageal separation. Compared to the Noggin secreted from the dorsal anterior foregut, that from the notochord has been proven more important for appropriate anterior foregut compartmentalization [33] because the knockout of *Nog* in the dorsal anterior foregut did not result in defects in mouse embryo mutants [33]. BMP signaling antagonism by Noggin is also crucial for the stratification of the squamous epithelium at the beginning of esophagus establishment [84]. Another study demonstrated that once the esophagus is well established, the maturation of esophageal basal cells requires limited activation of BMP signaling, while the inhibition of Bmpr1a was shown to downregulate the expression of suprabasal markers in these cells [85].

### 4.2. Wnt/β-Catenin Signaling

The canonical WNT pathway is implicated in the translocation of β-catenin to the nucleus [86]. The activation of Wnt/β-catenin signaling plays a crucial role in inducing proximal respiratory organs’ development and morphogenesis during embryogenesis, despite several studies demonstrating that the activation of WNT signaling seems more important in the generation of distal airway epithelium than proximal airway epithelium [86,87,88]. The occurrence of diseases including abnormal airway development and pulmonary inflammation has been proven to be related to the disruption of the WNT signaling [86]. Wnt/β-catenin signaling has been demonstrated to precisely regulate the tracheoesophageal separation. The early activation of Wnt/β-catenin signaling in endoderm is necessary to induce the respiratory fate on the ventral side of the anterior foregut endoderm, which is indicated by the expression of NKX2.1 (Figure 2C,D) [34,35]. However, on the dorsal side, the activation of WNT signaling by the presence of β-catenin induces the abnormal expression of Nkx2.1 in the esophagus, and the ligand Wnt5A inhibits the esophageal fate by downregulating the expression of Sox2 (Figure 2C,D) [16,63]. WNT signaling also plays an important role in respiratory mesodermal development by modulating the expression of TBX4 (Figure 2D). In mouse embryos, blocking WNT signaling with mutations in the *Ctnnb1* gene encoding β-catenin can cause the absence of Tbx4 expression, indicating loss of the respiratory fate in the mesoderm [34]. However, it seems the WNT-Tbx4 mesodermal pathway is restricted in tracheal mesoderm development but is not involved in lung mesoderm development [34]. Furthermore, the modulation of Wnt/β-catenin signaling affects the crosstalk between the anterior foregut endoderm and mesoderm. Without Wnt/β-catenin signaling from the tracheal epithelium towards the mesenchyme, the respiratory fate is maintained in the epithelium but not in the mesenchyme [34,89]. Excluding the possibility that Nkx2.1 from the tracheal epithelium induces the respiratory fate of the adjacent mesenchyme, a study observed the loss of Tbx4 expression in mesoderm, indicating the disappearance of the respiratory fate, after blocking WNT signaling in the tracheal epithelium [34]. Other studies tried to block another WNT ligand, Wls (also known as Gpr177), and observed the same result (Figure 2C,D) [89]. In the tracheal mesoderm, the loss of AXIN2, a scaffold protein in the β-catenin degradation complex, which is the gold standard reflecting WNT activation, affects the cartilage and overgrowth smooth muscle, different from healthy muscle organization [86,89]. Barx1, a transcription factor in dorsal anterior foregut mesenchyme, has also been revealed to mediate Sfrp1/2 to inhibit WNT signaling to induce the esophageal fate, and deletion of *Barx1* switches the esophageal epithelium to a respiratory fate (Figure 2B) [90].

### 4.3. Sonic Hedgehog (SHH) Signaling

*SHH*, a homologue of Drosophila Hedgehog, is a gene widely expressed in vertebrates [91]. This gene encodes multiple signaling molecules critically involved in the development and compartmentalization of the anterior foregut, airway morphogenesis, and lung development [91,92]. Loss of *Shh* in the foregut has been shown to induce the abnormal pseudo-stratification of the foregut epithelium [93]. During the separation between trachea and esophagus, Shh expression is present in both tracheal diverticulum from the ventral anterior foregut and in cells on the dorsal side, but the higher level in dorsal anterior foregut endoderm is required for generating mesenchymal force on epithelium during the physical separation and can improve the esophageal fate (Figure 2) [49,92]. The normal separation between the esophagus and trachea was not observed due to the deletion of *Shh* in mouse embryo mutants. Until E17.5, a single tube showing respiratory characters was connected to the stomach [92]. However, Shh expression is present in the developing esophageal endoderm after tracheoesophageal separation. Although Shh is expressed evidently on the ventral side before the separation, it is almost negative in E11.5 trachea [92]. However, during later stages, Shh signaling is activated between E13.5 and E15.5 in mouse trachea [92]. Furthermore, Shh from endoderm is important to induce the differentiation and proliferation of airway mesenchymal cells, including the cartilage progenitors with expression of Sox9 (Figure 2C,D) [92,94,95]. This induction has been shown to be dose-independent; different levels of Shh signalling from the epithelium leads to different regionalization of the gut tube mesenchyme [3,92]. In separated trachea, the mesenchymal cells not receiving Shh signals from epithelium cannot specify into cartilage leading to tracheomalacia [24,94,95,96]. Furthermore, patients with abnormal SHH signaling, such as GLI3-related Pallister-Hall syndrome showed laryngo–tracheal clefts [97]. The Shh signals from the esophageal epithelium are also decisive for the distribution of the esophageal fate to the adjacent mesenchyme and the smooth muscle development is disrupted in mice deficient in *Shh*, leading to megaesophagus with less proliferation of smooth muscle [98].

The dysregulation of SHH signaling has been proven to be related to abnormal anterior foregut organ development. Esophageal atresia with tracheoesophageal fistula (EA/TEF) and abnormal lung development were developed in homozygous *Shh*-null mutant mice [92]. Furthermore, a higher mesenchymal cell proliferation caused by abnormal Shh overexpression can result in death of the pups because of the lack of normal alveoli [99].

### 4.4. FGF Signaling

FGF signaling, which is principally from the mesenchyme towards the epithelium, is required for inducing the ventral side of the anterior foregut into the respiratory fate [100,101]. FGF2, secreted from adjacent cardiac mesoderm, contributes to the ventralization of human and mouse definitive endoderm, promoting the respiratory fate of anterior foregut including lung, trachea, and thyroid in a concentration-dependent way (Figure 2C,D) [102]. The exogenous treatment of FGF2 and FGF1 improves the proliferation of the airway epithelium and the maintenance of the level of other FGF signals, such as FGF7 during branching morphogenesis [103]. However, the normal development of the mouse esophagus after the separation still requires a low level of FGF signals and the excessive FGF signals secreted from the surrounding esophageal mesenchyme induce the respiratory fate [101,104]. Fgf10 has also been proven important in supporting the maturation of the respiratory mesenchyme (Figure 2D) [100,101]. Insufficient Fgf10 in lung mesenchyme affects the differentiation and the proliferation of smooth muscle and cartilage progenitors in the trachea [105]. Consistently, expression of Fgf1 and Fgf2 is localized in the mesenchyme of early mouse foregut [106]. Furthermore, Fgf10 negatively regulates Sox2 expression in the ventral epithelium (Figure 2C,D) [100]. FGF2 treatment has also been demonstrated to increase the expression of ventral anterior foregut marker, NKX2.1 in hESC-derived definitive endoderm culture, indicating that high FGF2 levels induce endoderm towards a respiratory fate rather than an esophageal fate [102]. Fgfr2 is a crucial FGF signaling receptor; its deactivation results in multiple malformations of digestive and respiratory system, including tracheomalacia and EA/TEF [101,104]. In rat embryos with EA/TEF, the expression of Fgfr2 is significantly reduced in the malformed fistula tract, suggesting the downregulation of the FGF receptor expression is associated with abnormal anterior foregut compartmentalization and respiratory-like tissue in the fistula [107].

## 5. Interactions Between Mesoderm and Endoderm During Anterior Foregut Organo-Specification Mediated by Coordination of Signaling Network

### 5.1. Dorsal-Ventral Patterning

During the development of the early foregut, a series of interactions between different signaling pathways leads to the specification of the ventral and dorsal domains of the anterior foregut, which give rise to the respiratory lineage [16,17,18,19,20]. These interactions induce the crosstalk between mesoderm and endoderm to improve this early regionalization before the tracheoesophageal separation. Retinoic acid (RA) induces the ventral specification of the lateral plate mesoderm and influences the adjacent endoderm by enhancing Shh expression. This loop permits Shh to activate the expression of Gli family in the ventral lateral plate mesoderm which is crucial in inducing the expression of Wnt2/2b and Bmp4 to improve and maintain the respiratory mesenchymal lineage [16,17,18]. Wnt2/2b expression mediated by β-catenin activation in the ventral mesenchyme induces the expression of Nkx2.1 in the endoderm, indicating the respiratory endodermal fate (Figure 2C,D) [16]. In parallel, endodermal BMP receptors responding to Bmp4 from the ventral mesenchyme inhibit the expression of Sox2 in the endoderm to prevent antagonism against Nkx2.1 expression and dorsal fate in the anterior foregut endoderm [18]. This circuit of signaling induces the ventral side of the anterior foregut into the early respiratory fate by inhibiting the expression of Sox2 and maintaining the esophageal fate on the dorsal side (Figure 2A,B) [16,17,18]. Following dorsal–ventral patterning, the interactions between BMP, WNT, SHH, and FGF signaling induce the midline endodermal constriction due to the compressive force from mesenchyme, and the separation between trachea and esophagus. In the ventral midline, Shh remains active to continuously stimulate GLI expression, whose disruption leads to the defects of foregut compartmentalization [17,92]. The activation of Shh is also required for the downstream expression of Bmp4 and Fgf10 in the ventral mesenchyme and for the precise positioning of the generation of tracheal primordium (Figure 2C,D) [17,108].

### 5.2. Tracheoesophageal Separation

During the remodeling of the epithelium at the separation process, the coordination between BMP and WNT signaling is still important to maintain the respiratory identity on the ventral side, by activating Nkx2.1 and inhibiting Sox2 expression [77,109]. Notably, the separation of epithelium is mediated by *Efnb2* and its loss results in failure of separation [64]. Wnt2/2b stimulates the response from the ventral endoderm to the Fgf10 secreted by the adjacent mesoderm, promoting the expression of co-factors and receptors required for ventral epithelial proliferation while inhibiting the dorsal fate (Figure 2C,D) [52,110]. Fgf10 from the mesenchyme stimulates the directional epithelial proliferation and outgrowth to start the tracheal primordium bulging on the ventral side of the anterior foregut [36,52]. This pathway is modulated by the different levels of Shh expression at different positions. The position with lower Shh secretion allows higher expression of Fgf10 and further promotes formation of the septum and initial budding [17,108]. WNT ligands such as Wnt5A promote Fgf10-directed ventral budding by regulating the cell polarity (Figure 2C,D) [18,109]. The physical separation of the dorsal and ventral anterior foregut initiates with the proliferation of epithelium, which is directed by the BMP downstream effectors including ERK. BMP modulates this process by influencing the tissue geometry and coordinating mechanical forces [18,77]. To enable a robust separation between trachea and esophagus, Shh modulates the positions of Fgf10 expression to limit the domains of the tracheal primordium outgrowth. BMP and WNT signaling continue coordinating to distinguish the respiratory and esophageal fate in the ventral and dorsal side of the anterior foregut, and their interactions with FGF signaling improve the appropriate proliferation of the epithelial cells [17,18,36,52,77,92,108,109,110].

### 5.3. Esophageal and Tracheal Maturation

Following the separation between trachea and esophagus, the specification and differentiation of the stratified squamous epithelium and the surrounding muscular structure are precisely modulated by the interacting network of signaling pathways (Figure 2) [22,111]. The continuous expression of Sox2 in mouse esophageal epithelium maintains the esophageal identity and together with the crosstalk between low-level BMP and WNT signaling, it improves the maturation of the esophagus [22]. However, this interaction is badly understood. Another study about osteoblasts has demonstrated that BMP-induced expression of Dkk1 improves the inhibition of canonical Wnt/β-catenin signaling [111]. The BMP signals from the mesenchyme, which are insufficient to switch the esophageal fate, may inhibit the activity of WNT in a similar way. This coordinative pathway is potentially involved in improving the specification and proliferation of basal cells, suprabasal cells, and stratified squamous epithelial cells [111]. In the esophageal mesenchyme, the skeletal muscle specification in the proximal part of the esophagus initiates from the smooth muscle differentiation. Myf5, which is a myogenic bHLH factor integrating with the BMP and FGF signals secreted from the surrounding mesenchyme, plays critical roles in this differentiation [112]. BMP signaling from the esophageal mesenchyme may be negatively regulated by its feedback regulator, SMAD6, to not disrupt normal esophageal epithelium stratification and mesenchymal patterning [113].

During the development of the trachea, this network of interacting signaling pathways is more complex, to induce appropriate epithelial and mesenchymal specification and proliferation. In mouse models, the canonical Wnt/β-catenin signaling remains active with Wnt2/2b expression to maintain the tracheal epithelial progenitor identity. Furthermore, Wnt signals activate Fgf10 in the adjacent mesenchyme, and receive feedback from Fgf10. This circuit between WNT and FGF signaling is efficient in preventing the premature epithelial differentiation to ensure sufficient proliferation of the undifferentiated progenitors [16,36,114]. Similar to the generation of tracheal primordium from the ventral anterior foregut, Fgf10 coordinates with Shh to regionalize the tracheal epithelium and mesenchyme [104]. The concentration of mesenchymal Fgf10 negatively modulates the epithelial expression of Shh signals during the epithelial cells’ expansion and this permits the Shh gradient to stimulate the expression of Sox9 and the differentiation of cartilage in the mesenchyme [95,104]. Furthermore, Shh induces Bmp4 expression in the mesenchyme to enhance the expression of Sox9 and the cartilage fate during this differentiation (Figure 2C,D) [115]. During specification of the tracheal epithelium, canonical WNT signaling improves the expression of respiratory epithelial transcription factors to induce early differentiation. Shh signaling is responsible for the specification of these epithelial cells by modulating the spatial expression of the transcription factors [16,89]. The coordination between WNT and SHH signaling ensures the proportion of the basal cells that will differentiate into different subtypes including ciliated and secretory cells [16,89]. The balance between Fgf10-Erk crosstalk and WNT activation is important for the specification of tracheal epithelium [101,116,117]. While the pseudostratified columnar epithelium becomes mature, the inhibition of the transcription factors crucial for the generation of ciliated cells is decreased by the coordinative reduction of Fgf10 and Erk, which improves the ciliation [117]. The fate of ciliated cells is also enhanced by the high activity of WNT in epithelium, with the improvement of FOXJ1 and MUC5AC expression [87,118]. Furthermore, non-canonical Wnt5A improves this process by adjusting the epithelial–mesenchymal alignment, cell polarity, and appropriate epithelium organization [115,118]. These interactions between FGF, SHH, WNT, and BMP form a self-regulating signaling network to induce multiple lineages in the tracheal epithelium and mesenchyme leading to the elongation and shaping of the trachea [16,89,114,115]. Some groups have also reported mesoderm–endodermal communication during the development of other organs from the anterior foregut and posterior foregut [119,120,121].

## 6. iPSC Models for Understanding Development of Anterior Foregut Organs

Given that animal models present limitations in the research of human embryogenesis and in investigations of the development of disease, more and more scientists are using human embryonic stem cells (hESCs) and human induced pluripotent stem cells (hiPSCs) as powerful *in vitro* tools (Table 1) [3,4,24,42,80,87,122,123,124,125,126]. Compared with animal models, using hiPSCs can better monitor the lineage trajectories and transcriptional programs during human anterior foregut organogenesis and disease initiation. Furthermore, hiPSCs and organoids are better for human preclinical tests, such as drug/toxicity predictivity [124,126]. Because of the pluripotent state of stem cells, they can be differentiated into organ-like cells, including respiratory-like and esophageal-like epithelial and mesenchymal cells [43,87,127]. Using a precise set of signaling factors/molecules which simulates *in vivo* regulation in hESCs and iPSCs to induce differentiation, differentiated cells or organoids are used to study the molecular signaling events during organogenesis and the development of disease [4,21,40,41,42,43].

### 6.1. Generating Endodermal Derivatives

**Figure 3:**
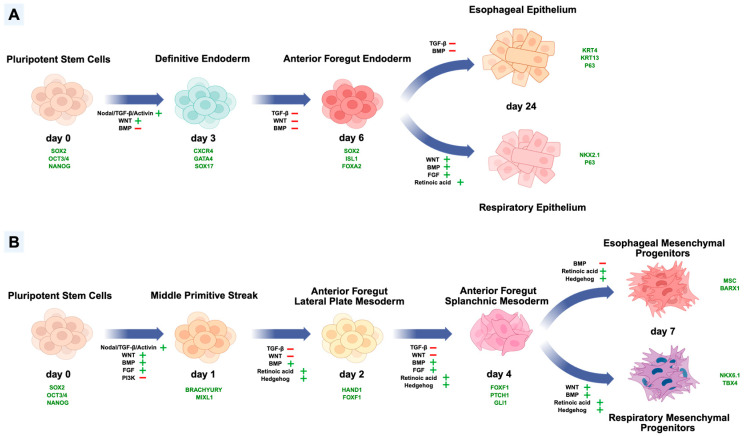
Pluripotent stem cell (PSC) differentiation into the esophageal and respiratory epithelium, and into the esophageal and respiratory mesenchyme. (**A**) Definitive endodermal cells (CXCR4^+^/GATA4^+^/SOX17^+^) are generated from PSCs (SOX2^+^/OCT3/4^+^/NANOG^+^) on day 3 following activation of Nodal and WNT signaling, while inhibiting BMP signaling. This is followed by induction of the anterior foregut endoderm (SOX2^+^/ISL1^+^/FOXA2^+^) on day 6 by inhibiting TGF-β, WNT, and BMP signaling. By day 24, esophageal epithelium (KRT4^+^/KRT13^+^/P63^+^) is generated by inhibiting TGF-β and BMP signaling, and respiratory epithelium (NKX2.1^+^/P63^+^) is generated by activating WNT, BMP, FGF, and RA signaling. (**B**) To generate the esophageal and respiratory mesenchyme, the middle primitive streak (BRACHYURY^+^/MIXL1^+^) is differentiated from PSCs on day 1 by activating Nodal, WNT, BMP, and FGF signaling and inhibiting PI3K signaling. The anterior foregut fate is induced in the lateral plate mesoderm (HAND1^+^/FOXF1^+^) on day 2 and in the splanchnic mesoderm (FOXF1^+^/PTCH1^+^/GLI1^+^) by day 4 by inhibiting TGF-β and WNT, and simultaneously activating BMP, FGF, RA, and Hedgehog signaling. By day 7, esophageal mesenchymal progenitors (MSC^+^/BARX1^+^) are generated by inhibiting BMP for 1 day and activating RA and Hedgehog signaling, and respiratory mesenchymal progenitors (NKX6.1^+^/TBX4^+^) are generated by activating BMP, RA, and Hedgehog signaling, with 1-day WNT activation.

The different methods of using stem cells to generate the esophageal and tracheal epithelium start with definitive endoderm induction [4,43]. Rodent studies demonstrated that the definitive endoderm is generated from the anterior part of the primitive streak [40,41,42]. Studies of hESC differentiation revealed that the simultaneous activations of BMP, WNT, and FGF for 1 day, combined with NODAL signaling activation, are critical for the generation of the primitive streak, in which lower BMP activation induced the formation of anterior primitive streak [44,45,131]. Miao et al. co-cultured endodermal and mesodermal cells to show that 24 h treatment of BMP4 is sufficient and appropriate to generate the anterior primitive streak and the definitive endoderm [132]. The prolonged duration of activation of BMP combining WNT can result in endoderm–mesoderm switching [42,132]. However, Matsuno et al. designed a simplified protocol for the induction of definitive endoderm after 1-day activation of WNT and 1-day inhibition of BMP, combined with persistent NODAL activation (Figure 3A) [133]. The generation of definitive endoderm can be confirmed by the expression of SOX17, CXCR4, and GATA4 [21,43,133]. After generating definitive endoderm on day 3, the next step is to generate anterior foregut endoderm. The combination of BMP inhibition and TGF-β from day 4 to day 5 followed by 1-day WNT inhibition can improve anterior foregut endoderm generation and esophageal specification [43]. The generation of anterior foregut endoderm can be confirmed by the expression of SOX2, FOXA2, and ISL1 [21,43]. To further differentiate the anterior foregut endoderm into esophageal epithelium, BMP signaling and the TGF-β pathway should be inhibited from day 6 to day 16, to pattern the endoderm to a dorsal fate, which is esophageal [43]. On the other hand, after differentiating endodermal cells into anterior foregut endoderm, WNT and BMP activation is necessary to induce respiratory fate [127,134]. Furthermore, combined FGF activation and RA treatment can improve the generation of NKX2.1^+^ airway progenitors (Figure 3A) [127,134].

After day 16, differentiated esophageal lineages are maintained in the culture to obtain mature esophageal epithelium containing esophageal progenitor cells (EPCs), which express FOXE1, P63, and PAX9 (Figure 3A) [43]. After day 24, Zhang et al. observed SOX9 expression, which is normally present in the human fetal esophagus, and the expression of KRT7, an intermediate filament protein that is expressed in the human fetal esophagus, but not in adults [43]. Therefore, the hPSC-derived EPCs generated from this procedure were confirmed to be like the esophageal progenitor cells during human fetal development [43]. These hPSC-derived EPCs were cultured in air–liquid interface, and the researchers observed the generation of a simple columnar epithelium which expressed KRT5 and P63 and the further development of stratified squamous epithelium containing different layers, a basal cell layer expressing P63, and suprabasal cells expressing KRT13 [43]. The generated stratified squamous epithelium was similar to the 10-week human embryonic esophageal epithelium, which was consistent with their observations in models of EPCs implantation under the kidney capsule [43]. This confirmed the potential of the hPSC-derived EPCs for simulating human esophageal morphogenesis, and this protocol of differentiating hPSCs into esophageal-like epithelium demonstrated the possibilities of mimicking the morphogenesis during esophageal epithelium development [43]. It also contributed a useful model to study the mechanisms of occurrence of the diseases, such as EA/TEF [21]. However, instead of generating anterior foregut endoderm, Trisno et al. induced anterior foregut spheroids from day 3 to day 6 and patterned the cells to the dorsal fate until day 9 [4,43]. They demonstrated that 1-day Wnt/β-catenin signaling activator treatment after the formation of definitive endoderm is crucial to generate the foregut spheroids and a simultaneous treatment of RA induced specifically anterior foregut patterning instead of posterior patterning, confirmed by the expression of anterior foregut transcriptional factors, HNF1β and SOX2 [4]. To dorsalize the anterior foregut spheroids in the next three days, the BMP signaling was inhibited by Noggin. Until day 9, they observed the cells of the generated spheroids expressed KRT4 and TP63 which specifically confirmed the dorsal fate [4]. These spheroids were maintained and became mature in one month. They showed the capability of the spheroids to proliferate into human esophageal organoids (HEOs) [4]. At 1 month, the size of HEOs had increased more than fivefold compared to day 9. Furthermore, the HEOs contained multilayered epithelium including P63^+^/KRT14^+^ basal and KRT13^+^ suprabasal cell layers, which coordinated to the composition of the EPCs induced by Zhang et al. [4,43]. Compared to mouse embryos, the composition of the cells of the HEOs at 1 month were similar to the esophageal epithelium at E17.5 [4]. The HEOs differentiated and proliferated the stratified squamous epithelium from 1 month to 2 months. The H&E staining images of 2-month HEOs showed more specified epithelial layers [4].

### 6.2. Generating Mesodermal Derivatives

Multiple studies have focused more on generating epithelium than mesenchyme to model diseases and *in vitro* differentiation from mESCs or hPSCs into esophageal and respiratory epithelium, developing a feasible way to model EA/TEF [21]. However, *in vitro* generation of epithelium is not able to replicate the complete process of development of the anterior foregut, esophagus, and trachea, which are modulated by the coordination between mesoderm and endoderm [3]. Therefore, it is necessary to also generate the esophageal-like and tracheal-like mesenchyme from stem cells and understand the molecular events during the development of anterior foregut mesodermal lineages whose dysregulation may lead to abnormal esophageal and tracheal specification. Generally, the differentiation from stem cells into mesodermal lineages starts with generating the middle primitive streak [3,38,42,44,45]. BRACHYURY, also known as TBXT, is an early mesodermal marker for the middle primitive streak and was generated by monitoring the activations and inhibitions during embryonic gastrulation. However, the traditional ways to generate middle primitive streak-like cells focused more on inducing early epithelial cells towards a mesodermal fate [79,135]. The activation of BMP signaling was proven crucial for the generation of mesoderm and the induction of epithelial–mesenchymal transition [38,45,79]. D’Amour et al. demonstrated that the duration of NODAL signaling affects the fate of the differentiating cells [131]. Longer activation of NODAL signaling results in an endodermal fate and activation that ended too early (after 24 h) generated mesoderm-like cells [131]. In fact, the use of Activin A is limited to generating the middle primitive streak in most of the existing differentiation protocols for mesodermal lineages [3,38,42,45]. Another group revealed that the prolongation of CHIR99021 treatment activating Wnt/β-catenin signaling improves the expression of Brachyury, inhibiting the endodermal fate at the same time [136]. Loh et al. generated highly pure BRACHYURY^+^/MIXL1^+^ human primitive streak-like cells from hPSCs after inhibiting PI3K signaling and activating WNT, FGF, and TGF-β signaling (Figure 3B) [45]. To mimic the development and specification of the mesodermal lineages after gastrulation, induction of a differentiated middle primitive streak leads to the generation of HAND1^+^/FOXF1^+^ lateral mesoderm, splanchnic mesoderm, and further organ-specific mesoderm [3,38,45,79,135]. Loh et al. specified the generation of lateral mesoderm and paraxial mesoderm by accurately regulating Wnt/β-catenin and BMP signaling [42]. The activation of BMP combined with the inhibition of Wnt/β-catenin signaling induced the primitive streak into lateral mesoderm, in contrast, paraxial mesoderm was elicited [42]. However, Kishimoto et al. improved Loh’s protocol to induce the anterior foregut mesodermal fate directly after generating the middle primitive streak [38]. During their differentiation from the middle primitive streak into lateral mesoderm, RA was added to improve the formation of splanchnic mesoderm including foregut mesoderm [3,38]. Furthermore, Hedgehog agonist was demonstrated to promote the anteriorization of the differentiating cells at the same time [38]. FGF was proven to compete with Wnt/β-catenin signaling to elicit the splanchnic mesoderm instead of limb formation [45]. The activation of FGF and BMP combined with TGF-β and WNT inhibition has become a common protocol to generate splanchnic mesoderm from lateral mesoderm in 2 days [3,38,45]. Kishimoto et al. continued RA and Hedgehog agonist treatment to keep the anterior foregut identity, and posterior foregut splanchnic mesoderm was also generated without Hedgehog agonist [38].

In Han’s protocol, the splanchnic mesoderm they generated is less specific; however, they improved organ specificity in the subsequent 3 days [3]. They successfully generated respiratory mesodermal progenitors by activating RA, Hedgehog, and BMP, with a low dose 1-day treatment of WNT activator. They also generated esophageal/gastric mesodermal progenitors using the same protocol by inhibiting BMP and without WNT activation [3]. Kishimoto et al. were able to generate more organ-specific mesenchyme, profiting from their strategy of an earlier inducing timepoint [38]. They derived esophageal mesenchyme and respiratory mesenchyme from anterior foregut splanchnic mesoderm, and liver-like fibroblasts and gastric mesenchyme from posterior foregut splanchnic mesoderm [38]. To induce the esophageal fate from anterior foregut splanchnic mesoderm, RA and Hedgehog are activated and BMP is inhibited for 1 day. In contrast, to mimic the embryonic tracheal mesenchymal development, BMP is activated followed by 1-day WNT activation, consistent with the tracheal induction of epithelium [38]. iPSC models create a specific platform to generate these different organ-specific mesenchymal lineages due to their common mesodermal origin along the anterior–posterior axis. However, the current protocols related to the generation of multiple types of specified mesenchymal cells could be improved. A study described successful differentiation to respiratory-like mesenchyme containing cartilage lineages and smooth muscle cells but could not generate more mesenchymal lineages such as airway fibroblasts [34].

These hPSC differentiation models provide platforms for understanding the abnormal development of the anterior foregut and tracheal–esophageal separation. A group has established *in vitro* model monitoring esophageal epithelium with EA/TEF, but the hPSC models mimicking tracheomalacia and laryngo–tracheal clefts still need to be created [21]. By using iPSCs derived from patients with EA/TEF and comparing with healthy-derived samples, Raad et al. observed the downregulation of SOX2 at the anterior foregut endoderm stage, indicating a dysregulated dorsal specification [21]. However, NKX2.1 expression was significantly higher in patient-derived esophageal epithelial cells [21]. In SOX2 knockdown models, anterior foregut endodermal samples derived from hPSCs, NKX2.1 expression was observed in the dorsal cell population [4]. Furthermore, it is necessary to generate SOX2 and NKX2.1 knockdown or epigenetic inhibition models based on techniques such as CRISPR. It is also important to include testing for upstream and downstream genes involved in BMP and WNT signaling via these models. Models using healthy-derived stem cells can mimic the pathogenic environment leading to abnormal esophageal and tracheal development such as EA/TEF. However, stem cells have not been widely used to investigate the molecular mechanisms in tracheal and esophageal mesenchymal development. Therefore, using hPSCs to understand esophageal and tracheal mesenchymal specification will be crucial for understanding the mechanisms leading to normal and abnormal tracheoesophageal specification.

## 7. Conclusions and Future Perspectives

**Figure 4:**
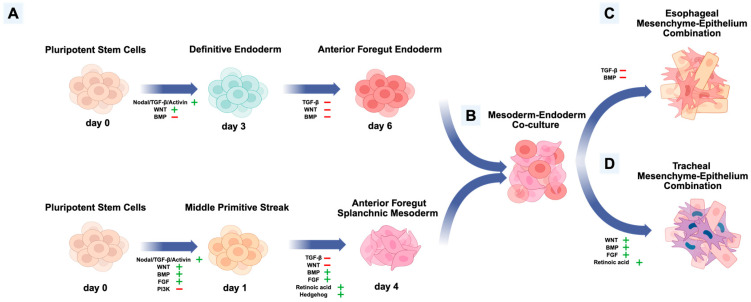
Schematic representation for generating Esophageal and Tracheal Mesenchyme–Epithelium Combination. (**A**) Simultaneous generation of anterior foregut endoderm and splanchnic mesoderm as described previously. (**B**) Co-culture of fixed proportions of anterior foregut endodermal and splanchnic mesodermal cells in media containing a cocktail of growth factors to induce organ-specific development, mimicking *in vivo* organogenesis. (**C**) By inhibiting TGF-β and BMP signaling, the generation of esophageal mesenchyme–epithelium is expected and (**D**) the generation of tracheal mesenchyme–epithelium is expected following activation of WNT, BMP, FGF, and RA signaling.

Due to the limitations arising from animal models, stem cell differentiation will become the principal method in understanding anterior foregut development, conditional upon appropriately controlling concerns including ethical issues, unpredictable mutations, and clonal variability of individual cell lines. Different protocols have been developed and improved based on previous understanding, to replicate important developmental stages of anterior foregut endodermal and mesenchymal lineages [3,38,42,45,79,87,122,123,135]. The anterior foregut endoderm differentiated from EA/TEF patient-derived iPSCs confirmed SOX2 reduction compared with healthy samples, and NKX2.1 respiratory fate has been observed in patient-derived esophageal epithelium [21]. With the protocol inducing mesodermal lineages, similar comparisons can be performed to understand healthy and patient-derived anterior foregut mesoderm and further esophageal and tracheal mesenchymal development. In the future, these comparisons will provide a map revealing the dysregulation during the induction of esophageal and tracheal fate, which may relate to abnormal responses to multiple signaling pathways. Genetic and epigenetic tools can be used on healthy-derived iPSCs to generate models mimicking a pathogenic environment, which will be useful for investigating rare malformations and developing new clinical therapeutics. Furthermore, the coordination between endoderm and mesoderm during anterior foregut development should also be monitored via *in vitro* studies [3]. Therefore, co-culture models including both endoderm and mesoderm will be useful for better understanding the molecular mechanisms in this stage. A co-culture can be created after the respective generation of early endodermal and mesodermal lineages, followed by the treatment of growth factors/molecules inducing organ-specific fates. The generation of esophageal or tracheal mesenchyme–epithelium culture or three-dimensional organoids will provide an innovative platform for understanding anterior foregut organogenesis (Figure 4). Combining strategies including cell sorting and niche modulation, we expect to generate more specified organ-specific mesenchymal and epithelial lineages from this platform, due to their interactions, which can better mimic natural organogenesis. Using micropatterned technology, Warmflash et al. integrated a mesodermal–endodermal structure by accurately adjusting pathway signals, whereby they revealed the effect of BMP4 on generating endoderm–mesoderm gradient in the same colony [79]. After 42 h of the BMP4 treatment, SOX2-positive cells were limited in the center of the colonies, with an outside ring-like mesodermal layer expressing Brachyury. Furthermore, EMT was confirmed in these colonies by the expression of SNAIL which is similar to the gastrulation in human development [79]. Another group derived mESCs into epiblast-like cells followed by activation of FGF, Nodal/TGF-β, BMP and WNT [135]. After 2 days, they successfully generated a well-organized early endoderm–mesoderm-like structure [135]. This indicates the possibility of generating cultures containing well-organized different germ layers from the beginning of the differentiation, which will better mimic *in vivo* embryogenesis. However, multiple difficulties exist in creating combined mesenchyme–epithelium culture. The proportion of each type of cells in the combination may influence the effect of recapitulating *in vivo* processes and the addition of different external inducing growth factors may affect or mask the natural interactions between different germ layers. Furthermore, the interactions between different organ lineages, such as those between the ventral anterior foregut endoderm and cardiac mesoderm, need to be better understood [102,119,137]. Models of abnormal development will be useful for determining genes related to multiple anterior foregut congenital diseases and correction of the phenotypes can be tested by correcting their expression using CRISPR/Cas technologies. Patient-specific iPSC differentiated cells could be used on artificial biomimetic constructs to replace damaged tissue or organs. Furthermore, therapeutics could be tested on patient-specific cells to deliver a more personalized medical approach. Microfluidic platforms can also be combined with iPSC differentiation to mimic the molecular gradient environment observed *in vivo* and generate more complex systems simultaneously containing epithelial and mesenchymal lineages with characteristics of different organs [138,139]. These deeper investigations of the complex molecular signaling cascades involved in normal and abnormal development will assuredly improve the therapeutic field in the future.

## Figures and Tables

**Table 1:** Comparison between animal models and iPSC-based systems describing their advantages, limitations and disease modeling relevance.

Dimension	Animal Models	iPSC-Based Human Systems
Advantages	a. Monitoring *in vivo* organogenesis [80] b. Causal pathway testing with *in vivo* genetic regulation [24,80]	a. Modeling human-specific early development and cell stages which may not be conserved in other species [80,128] b. Ability to create *in vitro* patient-specific developmental environment [129] c. Supporting isogenic controls to attribute phenotypes to specific variants [125]
Limitations	a. Difficulties in translation of gene regulation to human because of species differences [126] b. Difficulties in reproducing human congenital phenotypes	a. Limited long-term maturation [124] b. Variability between different cell lines and clones c. Missing systemic environment (e.g., immune cells and vascularization, etc.) [124,130]
Disease-modeling relevance	Defining developmental mechanisms and testing causal nodes related to diseases which need to be tested in human [24]	a. Best for *in vitro* human disease modeling due to recapitulation of patient-specific development [21] b. Manipulating healthy iPSCs using CRISPR/Cas to mimic diseases [4]

## Data Availability

The authors declare no data were used in this review.
